# Immunotherapy in the Context of Aortic Valve Diseases

**DOI:** 10.1007/s10557-024-07608-7

**Published:** 2024-07-17

**Authors:** Francesca Bartoli-Leonard, Tim Pennel, Massimo Caputo

**Affiliations:** 1 Bristol Medical School, Faculty of Health Sciences, University of Bristol, Bristol, UK; 2 Bristol Heart Institute, University Hospital Bristol and Weston NHS Foundation Trust, Bristol, UK; 3 Chris Barnard Division of Cardiothoracic Surgery, University of Cape Town, Cape Town, South Africa

**Keywords:** Aortic valve disease, Immune response, Calcification, T cells, Cardiovascular disease

## Abstract

**Purpose:**

Aortic valve disease (AVD) affects millions of people around the world, with no pharmacological intervention available. Widely considered a multi-faceted disease comprising both regurgitative pathogenesis, in which retrograde blood flows back through to the left ventricle, and aortic valve stenosis, which is characterized by the thickening, fibrosis, and subsequent mineralization of the aortic valve leaflets, limiting the anterograde flow through the valve, surgical intervention is still the main treatment, which incurs considerable risk to the patient.

**Results:**

Though originally thought of as a passive degeneration of the valve or a congenital malformation that has occurred before birth, the paradigm of AVD is shifting, and research into the inflammatory drivers of valve disease as a potential mechanism to modulate the pathobiology of this life-limiting pathology is taking center stage. Following limited success in mainstay therapeutics such as statins and mineralisation inhibitors, immunomodulatory strategies are being developed. Immune cell therapy has begun to be adopted in the cancer field, in which T cells (chimeric antigen receptor (CAR) T cells) are isolated from the patient, programmed to attack the cancer, and then re-administered to the patient. Within cardiac research, a novel T cell–based therapeutic approach has been developed to target lipid nanoparticles responsible for increasing cardiac fibrosis in a failing heart. With clonally expanded T-cell populations recently identified within the diseased valve, their unique epitope presentation may serve to identify novel targets for the treatment of valve disease.

**Conclusion:**

Taken together, targeted T-cell therapy may hold promise as a therapeutic platform to target a multitude of diseases with an autoimmune aspect, and this review aims to frame this in the context of cardiovascular disease, delineating what is currently known in the field, both clinically and translationally.

## Introduction

Aortic stenosis (AS) is a progressive obstructive disease of the aortic valve that increases the resistance across the left ventricular outflow tract. In its severe form, increased afterload results in left ventricular dysfunction, symptoms of left ventricular failure, and ultimately death [1]. The mechanism of this hemodynamic dysfunction within the valve [2, 3] occurs through osteogenic differentiation of valvular interstitial cells; calcification develops, culminating in calcific aortic valve disease (CAVD) [4–8]. Once mild valve obstruction is established, disease progression is inevitable, with surgical intervention as the only course of treatment once a patient is symptomatic [8–10]. Although previously thought to be a passive, degenerative pathology that occurs as a result of aging, aortic fatigue, or systemic deterioration of the matrix within the valve, it has more recently been demonstrated to be a result of a paradigm shift, suggested to be an active and cellular driven disease; however, treatment modalities have not yet caught up with this dogma [11–14]. With AVD representing a substantial and ever-increasing disease burden on the aging population, CAVD and subsequent AS are expected to increase from 2.5 million cases to 4.5 million by 2030 [15], and thus, there is now significant urgency for developing non-invasive treatment strategies for this ever-growing pathogenic population.

The diagnosis of AS is challenging when asymptomatic, and as such, most patients first present in its severe form with end-stage disease, where intervention is increasingly challenging and at times palliative. Due to the insidious nature of the symptoms of AS, such as decreased exercise tolerance, dyspnea, chest pain, and dizziness, patients often do not recognize the gradual limitations as a symptom, resulting in patients often not self-reporting. Thus, recent advances in echocardiography-based diagnostic techniques for valvular pathology are critical in diagnosing pathogenesis early [2, 16, 17], though stratifying which patients receive this beneficial diagnostic test has no current consensus [15]. It is known, however, that aging significantly correlates with moderate to severe symptoms of AS, producing yet another barrier in treatment strategies, with aged patients at higher risk for mortality during and directly after surgical intervention [18].

The current mainstay for treatment of symptomatic AS is aortic valve replacement (AVR) either by open surgery (SAVR) with a mechanical or bioprosthetic valve, transcatheter aortic valve replacement (TAVR), or more rarely balloon valvuloplasty [2, 19–23]. Surgical replacement is still considered the gold standard, though valvuloplasty does however offer a shorter relief for palliative patients and contraindications to SAVR or TAVR [22, 24, 25]. Conversely, balloon valvuloplasty still has a role in pediatric patients, allowing for more growth in the child before a definitive surgical valve replacement [25]. While the ideal valve prosthesis would be completely biocompatible without the need for anticoagulants or a limited lifespan [26], the current options are sub-optimal implants available, all with their own limitations and advantages. Limitations include but are not limited to coagulation complications with mechanical valves, a limited lifespan, degradation, and the need for reintervention with bioprosthetic valves [20, 27, 28]. Although bioprosthetic valves do not require anticoagulation and exhibit outstanding hemodynamics even in small roots/annulus, they lack the durability of mechanical valves.

While AVR remains the gold standard treatment therapy for symptomatic disease, deficiencies in the current devices available lead us to explore the biology of AS as a potential therapeutic solution to prevent progression to symptomatic severe disease. Thus, this review will outline the immune-driven degenerative process, highlighting where key immune cells come into play, before outlining potential immunomodulatory therapeutics, which should be considered for long-term treatment in aortic valve disease.

## Immune Cells Within the Aortic Valve

### Resident Immune Cells

Classically, aortic valves consist predominantly of two resident stromal cell populations: aortic valve interstitial cells (VICs) and aortic valve endothelial cells (VECs), the latter of which act as the interface of the valve and circulating blood [29, 30]. VICs are fibroblast-like cells derived from the cardiac neural crest, represent the dominant phenotype within the valve, and are responsible for the pathogenic calcification occurring during disease progression [31]. Layered within the extracellular matrix, VICs lay down further matrixes during development and homeostasis, which may provide an anchor to infiltrating immune cells.

Though the valve remains relatively acellular, the presence of leukocytes within the healthy aortic valve has been described. Hematopoietic marker CD45 + can be seen on 10–15% of cells within the valve [32, 33], with this fraction increasing as the patient ages [34]. The majority of CD45 + cells have been identified as surveilling antigen-presenting cells (APCs) which present antigens to the adaptive immune cells, known as either dendritic cells or macrophages, eliciting a targeted response. However, recent studies have suggested the presence of tissue-resident B and T cells within the valve [12, 35], although due to their suggested low population number and difficulty of obtaining healthy valve tissue, this remains largely unconfirmed. APCs, more frequently identified as macrophages than dendritic cells within the valve [12, 33], are responsible for the majority of low-level inflammation occurring within the healthy valve.

Sub-analysis of the CD45 + cells confirmed the presence of a major histocompatibility complex (MHC) class II + CD11c + CD86 + dendritic cell–like population within the healthy valve and in atherosclerosis [36–39]. CD11c + and CD86 + work in conjunction to co-stimulate T cells with MHC II antigen presentation, propagating T-cell activation [40, 41].

Secondly, CD68 + and CD206 + macrophages, previously described as M2, which are characterized as being responsible for wound healing and tissue repair have been identified within healthy valves and notably reduced in calcified valves [40]. The vast heterogeneity of tissue-resident macrophages suggests their multi-faceted role within the valve and may have originated during embryonic development, rather than by monocyte recruitment in an uninjured setting [42]. Suspected to be resident within the valves during development to clear apoptotic cells in the embryonic valves during the mid-late gestational period, cellular RNA sequencing studies have identified upregulation of immune activity in this gestational period [43, 44], and macrophages may remain in situ following birth. Conversely, murine models with the genetic depletion of endocardial-derived macrophage resulted in increased apoptotic bodies within the valve, suggestive of insufficient clearance, leading to thickened vales and remodelling [45].

Within the non-pathological valve, immune cells may act as surveillance cells, able to phagocytose pathogens and traffic to the lymphatic system, consistently being replaced and replenished [36], and may contribute to the local paracrine cytokine signaling within the valve, maintaining homeostasis.

### Infiltrating Immune Cells

**Fig. 1:**
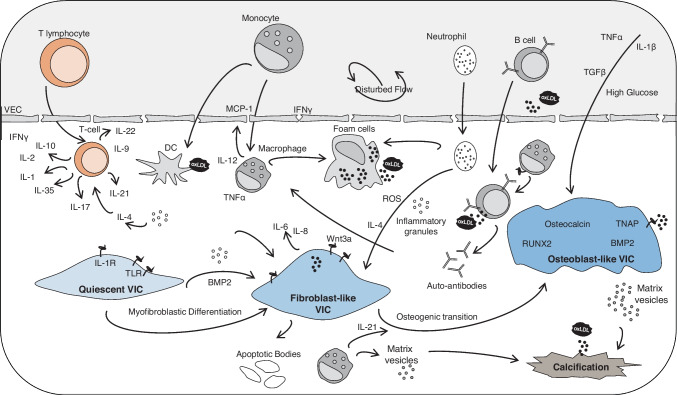
Role of immune cells in aortic valve disease. Complex immune cell networks play a crucial role in aortic valve disease. VECs under disturbed flow express adhesion molecules, allowing immune cells to infiltrate the valve. Adaptive immune cells, T lymphocytes and B cells, infiltrate the valve through the damaged endothelium. T-cell differentiation leads to the expression of both pro- and anti-inflammatory cytokines depending on the T-cell subtype. B cells respond to oxLDL through interaction with APC and produce pro-inflammatory cytokines and auto-antibodies, promoting further inflammation cascades. APCs, dendritic cells and macrophages, differentiate within the tissue from circulating monocytes. Both lipid and apoptotic body antigens are present within the valve, promoting an adaptive immune cell response and proliferation. Neutrophils enter the valve and produce inflammatory cytokines, reactive oxygen species, and cytotoxic granules, promoting cellular apoptosis and inflammation. APC, antigen-presenting cells; BMP2, bone morphogenic protein-2; DC, dendritic cell; IFNγ, interferon-gamma; MCP-1, monocyte chemoattractant protein-1; oxLDL, oxidized low-density lipoprotein; ROS, reactive oxygen species; RUNX2, runt-related transcription factor-2; TGFβ, tumor growth factor-beta; TLR, Toll-like receptor; TNAP, tissue non-specific alkaline phosphatase; TNFα, tumor necrosis factor-alpha; VECs, valvular endothelial cells; VIC, valvular interstitial cell

Similar to atherosclerosis and coronary artery disease, AVD has been suggested to be driven by inflammatory mechanisms, which promote the induction of pro-fibrotic and osteogenic pathways within the valves (Fig. 1). Both innate and adaptive immune cells are present in diseased valves, with infiltration by T lymphocytes suggested to be a critical driver of pathogenesis [12, 33, 35].

Through changes in the hemodynamic forces across the valve, VECs undergo phenotypic switching, leading to endothelial dysfunction leading to expression of adhesion molecules [46]. Propagated by the attraction of immune cells to the damaged and now exposed fibrotic matrix behind the VECs, immune cells produce pro-inflammatory cytokines and promote expression of endothelial nitric oxide synthase (eNOS) within the valve [47, 48], demonstrated to be increased in pathogenic valves. Through the increase in eNOS and subsequent oxidative stress, the valve moves in a positive feedback loop into further damage and inflammation, until surgical treatment is sought [49–51].

Peripheral-derived monocytes often characterized as short-lived circulating cells can differentiate into both DCs and macrophages following activation through polarizing markers within the valve [40]. Classical monocytes, CD14^hi^ and CD16^null^, comprise 90% of circulating monocytes and are strongly implicated in the propagation of pro-inflammatory signals releasing both TNFα and nitric oxide perpetuating an inflammatory environment [52–54]. Conversely, CD14^lo^ and CD16^hi^ non-classical monocytes constitutively produce IL-1RA and promote a reparative phenotype when differentiating into macrophages and surveilling the endothelium. In severe aortic valve stenosis and valve calcification CD14^hi^, CD16 + intermediate monocytes have been shown to be present, expanding from a negligible population to up to 8% of the circulating monocytes [32, 53, 55–57]. Exhibiting large amounts of reactive oxygen species and inflammatory markers, interferon-gamma (IFNγ) intermediate monocytes represent the greatest proponent of chronic inflammation within the valve of all monocytes.

Once monocytes have infiltrated the tissue, they differentiate into macrophages or dendritic cells. Macrophages have been consistently associated with the progression and severity of atherosclerosis [58–61] and identified in stenotic and calcified aortic valves [12, 32, 62]. Following endothelial dysfunction within the valve, adhesion markers are upregulated allowing for increased monocyte infiltration and remodeling of the extracellular matrix, propagating further inflammation. Pro-inflammatory macrophage expression-induced nitric oxide synthase (iNOS) TNFα, IL-6, IL-12, and MCP-1 serve to attract further pro-inflammatory cells to the region and propagate the inflammatory cascade [12, 63]. Notably conditioned media taken from pro-inflammatory macrophages can polarize VICs to a fibro-calcific phenotype, giving further evidence to their pathogenic role in disease progression [62, 64, 65]. Oxidized LDL (oxLDL) present in a high lipid environment conducive to AVD promotes monocyte maturation, adhesion to the ECM, and the differentiation of macrophages to foam cells through scavenger receptor lipid uptake [66–69]. Foam cells release pro-inflammatory markers IL-1β and TNFα, while inhibiting IFNγ-induced inflammation. The terminal differentiation of macrophages into foam cells limits the cell’s ability to phenotypically shift to an anti-inflammatory phenotype [59, 66, 70–72], as they continue to promote osteoclastogenic activity through RANKL activation and inhibit osteoblast formation.

B cells are distinct from other immune cells, with their ability to respond to both self and foreign antigens through the immunoglobulin B-cell receptors. Through atherosclerotic studies, B cells have been demonstrated to promote a pro-atherogenic response through secretion of granulocyte–macrophage colony-stimulating factor (GM-CSF) which promotes the polarization of pro-inflammatory monocytes/macrophages to the localized area [57, 63, 73]. The pro-inflammatory environment of the valve enhances the pathogenic switching of B cells from producing IgG to IgE, the latter of which has been associated with echocardiographic markers of aortic stenosis severity [74]. Through the promotion of macrophage localization, B cells have been suggested to be implicated in the progressive thickening of the valves, contributing to pathogenic degradation [75–78].

T lymphocytes have been shown to promote pathological remodeling during coronary disease and atherosclerosis in chorus with macrophages [60, 79, 80]; however, their role in AVD pathogenesis is still misunderstood. Utilizing in vivo imaging, cell lineage tracing, and murine models, T cells have been identified as critical drivers of pathogenesis in atherosclerosis [39, 81–83], further suggesting a possible cross-over with calcific valve degeneration [12, 51, 84–86]. First described through histology as co-localizing with IL-2, T lymphocytes were believed to be skewed toward a CD4 + rather than CD8 + subset, with cytotoxic T-cell infiltrates aggregating around regions of calcification and dysfunction [87]. In end-stage aortic stenosis, T cells have been co-localized around neoangiogenic regions, indicative of an immunomodulatory process and the culmination of chronic remodeling within the valve [88]. CD4 + T cells have the capacity to differentiate into both T helper or T regulatory cells, dampening down the response through physical interactions and releasing cytokines to other immune cells [89]. Releasing TNFα and CD4 + T helper cells has been shown to enhance mineralization matrix nodule synthesis through the promotion of osteogenic VIC differentiation and the sequestering of pro-fibrotic enhancers in the valve [17, 51, 85]. Pro-inflammatory Th17 CD4 + T cells promote calcification and pathogenesis through TGFβ release and apoptosis of neighboring cells through IL-6 and IL-21, promoting fibrosis and mineralization through apoptotic-driven mechanisms [90, 91]. Moreover, IL-21 promotes osteoblastic differentiation in VICs through the upregulation of alkaline phosphatase and RUNX2, further promoting calcification within the tissue [92]. Notably, the hallmark of Th17 and IL-17 has been shown to correlate with the severity of hypertension and atherosclerosis and seen in CAVD patient plasma correlating with disease progression [93]. Conversely, regulatory T cells (Treg) promote immunologic tolerance within tissue, suppressing pro-inflammatory responses and reducing IgE production. Treg populations have been found within severe aortic stenosis, with little understanding of whether this is a resolving response or a response to the propagation of further inflammation [51, 89, 94]. CD8 + T cells also known as cytotoxic T cells play a critical role against intracellular pathogens through the secretion of pro-inflammatory cytokines, the utilization of the FasL to bind and promote apoptosis within the target cell, and finally the release of serine-protease granzymes and perforin to breakdown and target the cell for apoptosis [56, 87, 95, 96]. Within aortic stenosis, CD8 + cells have been suggested to contribute to disease progression, through the impairment of osteoclast function, reducing the cell’s ability to reabsorb calcium from the valvular matrix [30, 96, 97]. More recently, clonal expansion within CD8 + T cells has been shown to occur within the tissue, rather than the peripheral, correlating with the trafficking of monocytes into the lesion. With the limited T-cell repertoire present in the valve and increased CD8 + population within late-stage disease, targeting these cells against specific pathogenic epitopes may be a viable option for immunotherapy to target aortic valve disease.

## Non-surgical Treatment of Aortic Valve Disease

Understanding the immunological process in valve disease may provide a fresh perspective on the treatment and future dissent into pathogenesis. Currently, no drug strategies in the treatment of AVD have proven effective in the clinic, leaving valve replacement the only option [98]. The use of lipid-lowering therapies has shown reasonable success in large-scale atherosclerotic studies [99–102], with a reduction of overall disease burden and increased life expectancy. With AVD correlating with dyslipidemia and early studies identifying substantial lipid accumulation in aortic leaflets [9, 14, 103–108], lipid-lowering therapies were believed to be a viable option in the reduction of calcific burden in AVD. While several preclinical studies have suggested that 3-hydroxy-3-methylgutaryl-coenzyme A (HMG-CoA), also known as *statins*, may reduce the calcific burden in AVD, clinical trials have not demonstrated a therapeutic benefit for this patient cohort. Notably, the *Scottish Aortic Stenosis and Lipid Lowering Trial, Impact on Regression* (SALTIRE) study [109] utilizing atorvastatin to reduce calcific aortic stenosis showed no benefit in improving outcomes or halting disease progression. Additionally, the *Aortic Stenosis Progression Observation: Measuring Effects of Rosuvastatin* (ASTRONOMER) trial [110] assessing the effect of rosuvastatin in asymptomatic patients with mild to moderate AS demonstrated no reduction in the progression of AS, giving further evidence to the limited efficacy in lipid-lowering therapies in aortic valve disease.

Immunomodulatory drugs have also shown limited efficacy in the treatment of calcific vascular disease, though limited studies have been conducted on the valve. Parallels between AVD and atherosclerosis, while mechanistically distinct, can be drawn. In regard to uncontrolled immune responses and chronic inflammation, atherosclerosis is initiated via activation of the endothelium layer, followed by a cascade of lipid deposition, fibrosis, and calcification, resulting in a narrowing of the vessel and a positive feedback loop of inflammation [38, 39, 59]. Conversely, lipid deposition and inflammation are the nuclei cause of AVD, resulting in a thickening, fibrosis, and calcification within the valve leaflets [9, 14]. While the pathogenesis differs, clinical trials have attempted to utilize similar therapies for both pathologies, to limited effects. Since the turn of the century, a multitude of clinical trials have attempted to utilize anti-inflammatory therapies for the reduction of atherosclerosis, none of which have shown marked success: oxLDL antibody, BI-204 to inhibit the uptake of oxLDL into macrophages [111], and losmapimod, p38-MAPK inhibitor to inhibit the differentiation of macrophages into foam cells have had limited success, in part due to either the insufficient inflammation present to sequester or the mechanistic redundancy in the trans-differentiation of macrophages to foam cells [112]. Alternatively, targeting secreted phospholipases A2 (sPLA2) which were shown to promote inflammation within atherosclerosis and increase in expression with calcified aortic valves [113] was attempted through the use of varespladib, atreleuton, and veliflapon, to limit the promotion of leukocyte recruitment within the vasculature [114–116]. All of these showed limited efficacy, suggesting that attempting to limit infiltration and propagation of cells might require a more targeted approach than predominately lipid-driven pathogenesis. Conversely, however, the Canakinumab Anti-inflammatory Thrombosis Outcome Study (CANTOS) demonstrated that anti-inflammatory drug targeting of interleukin 1-beta led to significantly lower rates of recurrent cardiovascular events [117], highlighting the critical role the immune response plays in pathogenesis.

## Immunotherapy as an Emerging Avenue for the Treatment of Aortic Valve Disease

As highlighted above, the pharmacological treatment of chronic inflammatory-driven pathologies such as AVD is critically lacking. While in vitro studies have shown the efficacy of a multitude of drugs [118–120], few have been able to replicate the success in vivo, suggesting the need for new approaches. While the potential for immune modulation to impact the heart has been appreciated for decades, targeting immune modulation in atherosclerosis has been heavily discussed [121, 122], but still not implemented fully within atherosclerosis or AVD. Recent work in the cardiac fibrosis field has highlighted the opportunity to engage cellular targeted approaches, through advancements in lipid nanoparticles carrying messenger RNA (mRNA) which can specifically prime and engineer T cells, or any other type of cell, to target specific markers [123–125]. While immune responses against foreign epitopes are similar to those raised against pathogens and act quickly to remove the infection, the ability of the endogenous T cells is limited, due to suppression by central or peripheral tolerance mechanisms, namely, through thymic elimination of high-affinity T-cell receptors to self-antigens, leaving a predominantly low-affinity self-responsive population [126]. Notably, T-cell receptors against self-epitopes have a 1.5 log lower affinity for cognate MHC:peptide complex, relative to the virus-specific TCR counterparts, suggesting an inherent inadequacy to remove self-epitopes [127, 128]. The recent development of engineering T cells allows the genetically targeted reprogramming of T cells with chimeric antigen receptors (CARs). CARs are synthetic receptors, which alter the ability of the T cell, now known as a CAR-T cell, to recognize pre-defined surface antigens with a higher degree of specificity in a non-MHC restricted manner, allowing the CAR-T cell to target any specific cell surface receptor in the cell membrane [129, 130]. With the known presence of T cells within both the healthy valve and the elevated infiltration during valve degeneration, CAR-T therapy would be capable of specifically targeting pathogenically activated VICs or pro-inflammatory cells with a unique membrane protein signature.

Identification of the appropriate marker to target, however, is a challenging question [131]. Unlike in cancer, where a specific mutation can be identified and targeted as the unique marker of cancer, linage-specific markers, such as bone morphogenic proteins, osteocalcin, and alkaline phosphatase, which are often associated with AVD cannot be used [6, 7, 12, 13], as targeting their normal counterparts expressing them will cause unacceptable side effects [132]. Thus, the best identification method of CAR-T targets may come from understanding the specific mutations within the individual patient through sequencing [123], for a personalized medicine approach to treatment.

Through the use of CAR-T has been standardized as a second line of defense in cancer treatments, CAR can in fact be applied to a range of immune cell–driven pathologies, giving them the signal to target specific antigens and initiate an antigen-specific immune response. Conventional CAR-T therapy involves the isolation of cells from healthy donors, genetically modifying the T cells ex vivo to target the surface protein found on a tumor cell, expanding the cell population, and then re-injecting the expanded cell population into the patient to target the tumor [129, 130]. The selection, expansion and reintroduction process can introduce unwanted allergic responses to the injected cells and with a reduction in the host immune system leaves the patient vulnerable to opportunistic infections. Seminal work in the blood cancer field has demonstrated the efficacy of these mechanisms, though using retroviral transduction to produce long-term CAR expression in the cells, lasting for months if not years within the patient [131]. Within the field of cancer, this long-lasting impact is precisely what is required to continue to sequester cancer cells as they grow, taking a persistent role in the body for long-term therapeutic efficacy. However, with the discrepancy in temporal profile in AVD compared to cancer, the requirement for persistent CAR-T activation is less. Thus, the long-term persistence of pathogenic cells within the valve may present a safety risk. Transient-engineered T cells, such as those generated in vivo, without the need to remove and reinject blood cells, offer a preferential alternative. A recent groundbreaking study developed a novel method to generate transient CAR-T cardiac targeting cells in vivo, bypassing the requirement for manufacturing and long-term safety of these cells [123, 133]. Through their approach, modified mRNA targeting a specific pathogenic marker is packaged into lipid nanoparticles (LNPs) encased in an antibody-laden membrane.

**Fig. 2:**
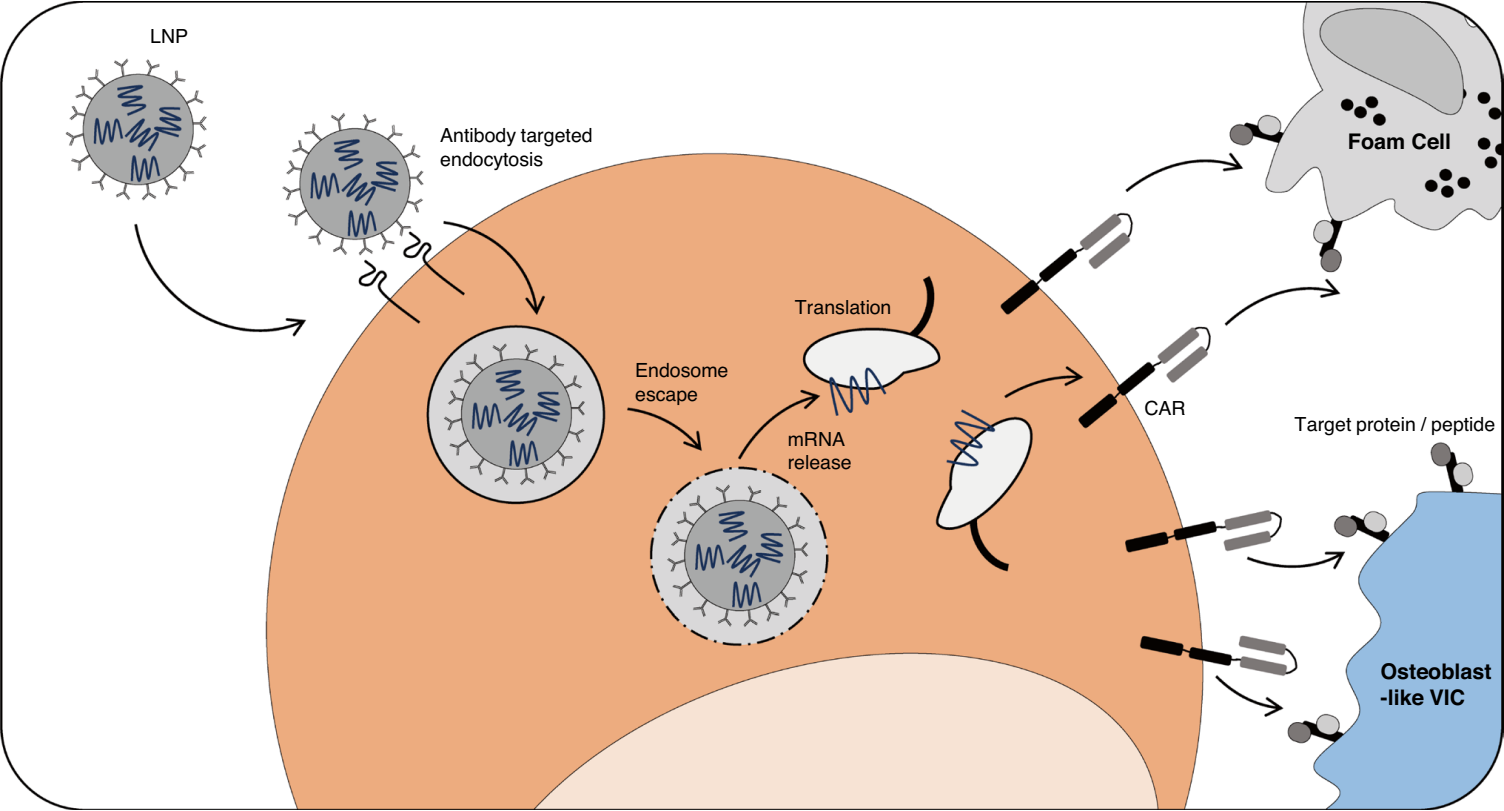
Proposed mechanism of CAR-T for aortic valve disease. Targeting resident T cells present in the valve against pathogenic aortic valve cells through the use of LNPs and known T-cell receptor targeting. Identification of a potential target may be identified through sequencing screening of a calcified tissue or liquid biopsy. LNP decorated with antibodies targeting T-cell surface receptors is endocytosed and encapsulated in an endosome within the T cell. Following endosome escape, mRNA is released into the cytoplasm and translated into a CAR, which is presented on the T-cell surface, specifically targeting cell surface protein on a target cell. LNP, lipid nano-particle; CAR, chimeric antigen receptor, VIC, valvular interstitial cell

These antibodies target uptake by a specific cell type, in this case, CD5, a T-cell surface protein which selectively activates T-cell endocytosis and is not required for T-cell effector function. Through endocytosis-driven release of the mRNA once taken up by the cells, the mRNA is translated to generate the CAR-T cells in vivo (Fig. 2). The transient stability of mRNA is well documented; mRNA is restricted to the cytoplasm of the cell, unable to undergo genetic integration and diluted during cell division, and the CAR expression within these T cells cannot be permanent. Within the murine model, a consistent CAR + T cell population was identified 48 h post-LNP injection, across each major T-cell subset, with a decline in a cell population within 10 days.

A similar methodology has also been implemented in the removal of senescent cells [134]. Similarly to the pathogenic cells within AVD, senescent cells do not divide or create an immunosuppressive environment; in comparison to cancer, this may present fewer barriers to the development of therapeutically efficacious CAR-T therapy. By targeting only senolytic cells rather than highly replicative cells, the risk of potential cascading effects of removing functional cells is limited, thus minimizing side effects. With the success of mRNA-LNP vaccines for COVID-19, the efficacy and safety of this mechanism of delivery have been extensively documented and thus present a viable path for the development of scalable immunotherapies. The use of these cells in a model of heart failure gives the first proof of concept that modified mRNA encapsulated in LNPs targeting T cells could be delivered to address a chronic cardiac pathology through the production of functionally engineered T cells in vivo. In comparison to cancer, where the presence of a single cell left behind can be devastating, AVD due to its slow pathogenic development would require a much lower dose, with the removal of 30–50% potentially able to restore adequate valve function. Critically, utilization of this therapeutic strategy would require comparing proteomic or gene expression profiles of a multitude of pathogenic and healthy valves to identify a target unique to AVD [132, 135]. The ideal antigens for engagement in CAR-T would be highly expressed on target cells, but not in vital tissues with a CAR signature unique so as to not mistarget. The heterogeneity and complexity of AVD may require a combination of antigens to target to deliver acceptable efficacy, with the potential for personalized medicine to play a leading role in the development of such precise therapy. Incredible progress has been achieved in the preclinical use of engineered T cells, and the ability to extend the use of these cells beyond oncology to one of the leading cardiac pathologies globally offers an exciting opportunity.

## Conclusions

Though advances have been made in the surgical interventional strategies for the treatment of aortic valve disease, pharmacological interventions are still lacking, highlighting the need for novel approaches to address the ever-growing clinical burden. Clinical trials utilizing statins and lipid-lowering therapies have demonstrated limited efficacy and highlighted the requirement for differing treatment strategies between valve and vascular pathologies, with the preservation of the dynamic valve hemodynamic environment critical for restoring the function of the valve. With the inclusion of multi-omics datasets and single-cell technologies to aid the understanding of the cellular and inflammatory landscape within the valve, targeted therapies such as immune-targeting lipid-delivered targets might offer such resolve in the future.

## Data Availability

Not applicable.
